# Lipoma of Columella with septal extension in Pai syndrome: report of a rare case

**DOI:** 10.1186/s12901-017-0035-y

**Published:** 2017-02-06

**Authors:** Surendra B. Patil, Shree Harsh

**Affiliations:** Department of Plastic and Maxillofacial Surgery, Government Medical College and Hospital, Nagpur, Maharashtra India

**Keywords:** Columella, Lipoma, Pai syndrome, Case report

## Abstract

**Background:**

Lipoma in nasal region in a case of Pai syndrome is very rare. Very few cases of Pai syndrome with columellar lipoma with extension to septum and tip have been reported till date.

**Case presentation:**

We report a very rare form of nasal lipoma in a case of Pai syndrome with extension to septum and tip in a 4 year old female child.

**Conclusions:**

Pai syndrome with columellar lipoma with septal and tip extension should always be kept in mind when evaluating a swelling in the region of nasal tip, collumella and septum with notch in upper lip and corpus callosal lipoma.

## Background

Lipoma is the most common mesenchymal origin tumor. They are commonly found in trunk, neck, upper and lower extremities. Head and neck region makeupto 13% of lipomas [1]. In the head and neck region, they occur most commonly in the posterior region. Lipoma in the nasal region is very rare. In the nose, lipoma has been reported in the tip [2], nasoalar crease [3], and in the vestibule [4]. We are presenting a case of nasal lipoma involving columella, septum and tip in a case of Pai syndrome.

## Case presentation

Parents of a 4 year old female child presented her with deformity of the nose and difficulty in breathing from right side of the nose for 1 year. She developed the deformity as a small swelling at the tip of the nose of about 0.5 cm which increased progressively to the size of about 1 cm in 1 year. There was no history of trauma, fever, sudden increase in size or pain associated with the swelling. There was no association of similar swelling in any family member. Child was immunised and had achieved milestones appropriate for her age. Parents of the child wanted removal of the swelling as it was a cause of social embarrassment for the child and she had difficulty in breathing from right nostril. The child had an identical twin who was operated for intususseption at 6 months of age.

**Fig. 1:**
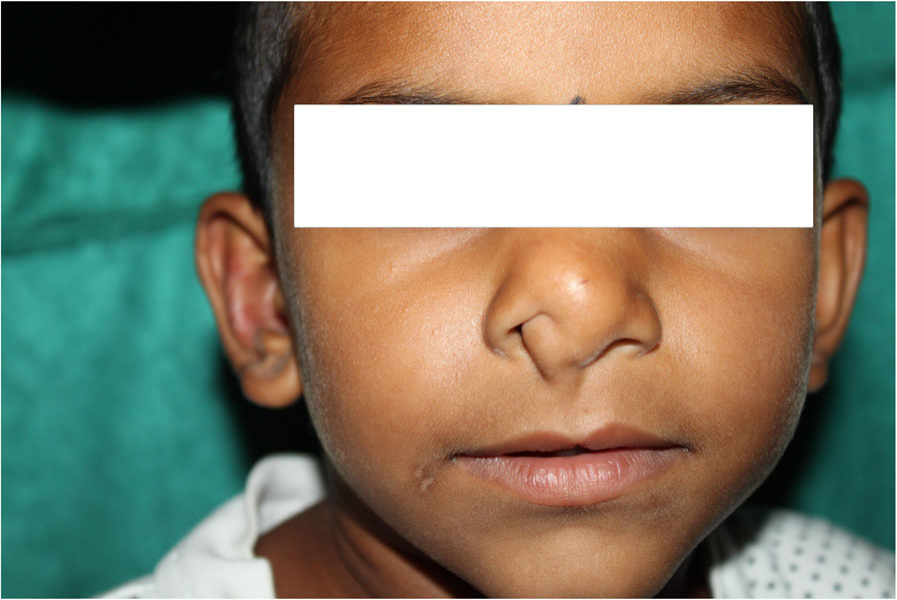
Preoperative photograph

**Fig. 2:**
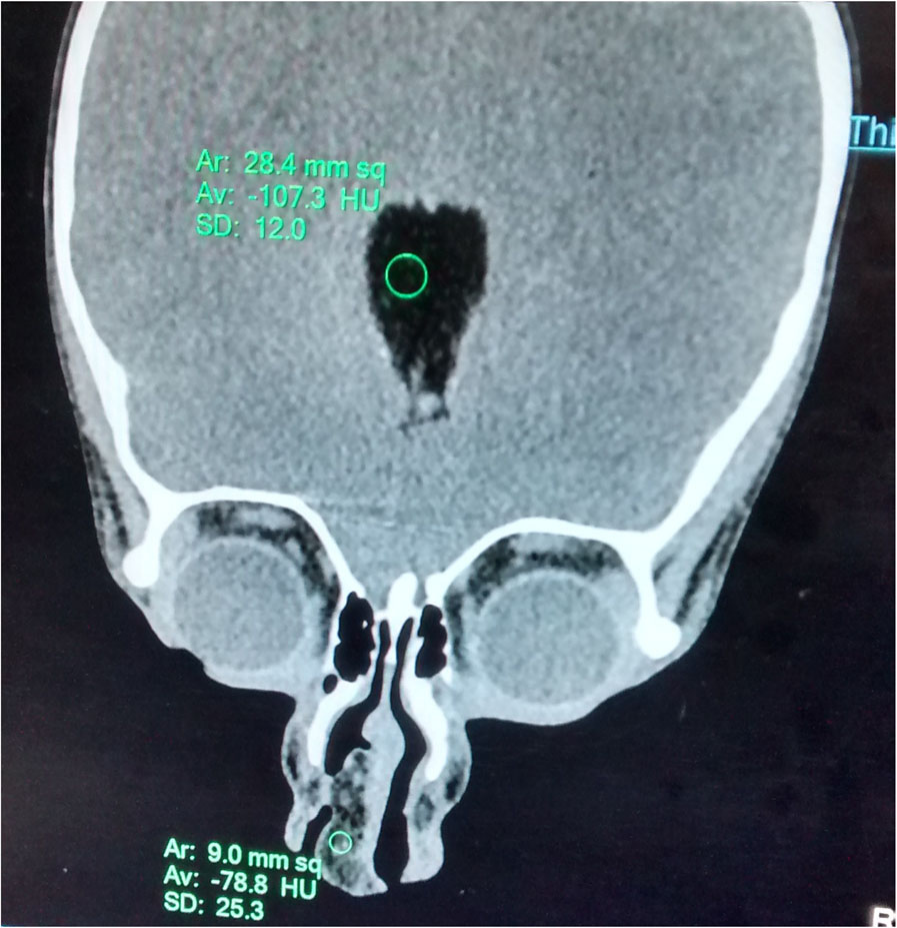
CECT Coronal view -Lipoma in the genu of corpus callosum and nasal tip with obliteration of right nasal cavity

On examination, there was evidence of a diffuse 1.2x1x1.5 cm swelling at the tip of nose in maximum horizontal, vertical and anteroposterior dimensions. The skin over the swelling was found to be normal and free from the swelling. It was soft in consistency. Swelling extended to the nasal columella. Right ala was found to be slightly flared. It was non tender. The septum appeared normal. There was midline cleft of the upper lip (Fig. 1). X ray chest showed levocardia. 2D echo was normal. Contrast enhanced Computed Tomography of para nasal sinuses and head showed a non enhancing fat density lesion of approximate size of 0.8x1.2x2.3 cm in the tip of nose and inferior nasal septum causing compromise of right nasal cavity (Fig. 2). There was an incidental finding of lipoma in the genu of corpus callosum with bracket calcification. Patient was posted for exploration of the swelling after anesthetic fitness and written consent from the parents.

Nose was opened with stair step incision in the collumella for open rhinoplasty. This incision was chosen over sublabial or septal incision in view of better operative view in the case of former incision. Fatty tissue was found over the left lower lateral cartilage. Right lower lateral cartilage was found to be deficient. Lipoma extended till the middle of collumella on the right of middle crus and right part of the caudal septum. It was excised in toto and specimen was sent for histopathological examination.

**Fig. 3:**
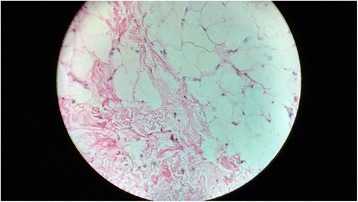
10 × H and E stain - Mature looking adipocytes and muscle fibers

**Fig. 4:**
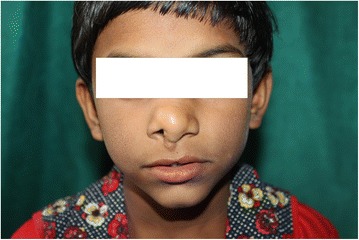
Postoperative photograph

**Fig. 5:**
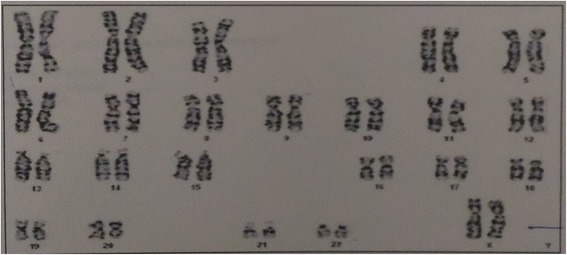
Karyotype

Postoperative period was uneventful and patient was discharged on the third postoperative day. Histopathology was suggestive of lipoma (Fig. 3). Early followup did not show any recurrence. There was a residual collumellar deformity which required secondary revision (Fig. 4). Patient’s patient did not wanted any surgery at that time. Karyotype analysis was normal (Fig. 5). She was advised further followup at regular intervals. Patient was satisfied with the surgical outcome.

## Discussion

Lipoma are found mostly in 5^th^ to 6^th^ decade of life [1]. It is the most common soft tissue tumor in adults. They are uncommon in paediatric population. Cases of nasal lipoma have been reported in the past in adults. There may be many causes of nasal swelling in paediatric age group. Differentials include abscess, polyps, posttraumatic deformity, hematoma and congenital anomalies like teratoma and hemangioma. Septal lipoma has been reported in paediatric population in a case of Pai syndrome and in neonate with fibrolipoma [5].

Many liopomas of the nasal and paranasal sinuses have been reported. Lipoma have been reported in oral cavity, hypopharynx, parotid, Retropharyngeal area and larynx [1]. Diagnosis is clinical in subcutaneous lipoma supported by fine needle aspiration cytology. It may be supplemented with computed tomography which shows homogenous low attenuation mass between −65 and −125 hounsfield units. Capsule of lipoma is not distinct.

Midline cleft of lip along with nasal polyps and corpus callosal lipoma has been described in Pai syndrome [6]. The syndrome was reported for the first time in 1987. Since then very few cases have been reported. It is a rare developmental anomaly of face. Median cleft of lip is rare and found in 0.2% of patients with orofacial clefts [6]. Classicaly Pai syndrome is associated with complete midline cleft of the upper lip, cutaneous polyps and central nervous system lipomas [7]. Hernia of Inguinal region, cryptorchidism along with clinodactyly have also been reported. Double frenulum of upper lip with median alveolar cleft [8] has been reported in Pai syndrome. It has been once reported in a twin [9] of Arabian descent.

Prenatal diagnosis can be done with the help of sonography and MRI [10]. Agenesis of corpus callosum with hemartomatous mass in nostril in Pai syndrome has been reported in a 1 month male child [11]. Hypopigmented fundi with pigmented rings in the optic disc and hypopigmented macule with severe midline cleft of lip and palate in a child of Arabian descent [12] has been reported from Qutar. Heriditary association of Pai syndrome and presentation of coloboma of right iris [13], with conjunctival lipoma of right eye [14], with bifid nose and frontal alopecia [15] and de novo reciprocal translocation have been reported [16]. Prenatal counselling following prenatal detection of pericallosal lipoma should be done [17].

## Conclusion

Nasal lipoma in pediatric age group in association with Pai syndrome in a twin is very rare. With proper knowledge of differentials and appropriate examination supported by cytology and imaging studies, diagnosis can be made. Surgical approach depends upon the position of the lipoma. Open rhinoplasty was the answer in our case.
